# Genetic diagnosis and clinical analysis of 17α-hydroxylase/17, 20-lyase deficiency combined with type 2 diabetes mellitus: A case report

**DOI:** 10.1097/MD.0000000000036727

**Published:** 2023-12-29

**Authors:** Yumin Zhang, Yuexing Yuan

**Affiliations:** a Department of Geriatric Endocrinology, Jiangsu Province Hospital and Nanjing Medical University First Affiliated Hospital, Nanjing, Jiangsu Province, China; b Department of Endocrinology, Zhongda Hospital, Southeast University, Nanjing, Jiangsu Province, China.

**Keywords:** 17α-hydroxylase/17, 20-lyase deficiency, case report, congenital adrenal hyperplasia, CYP17A1, type 2 diabetes mellitus

## Abstract

**Rationale:**

17α-Hydroxylase/17, 20-lyase deficiency (17OHD) is a recessively inherited autosomal disease caused by CYP17A1 gene mutations. It is characterized by failure to synthesize cortisol, adrenal androgens and gonadal steroids. However, it is rare in clinic combining with type 2 diabetes mellitus (T2DM).

**Patient concerns:**

A 21-year-old woman was transferred to an endocrinology clinic because of paroxysmal paralysis. In addition, she presented with hypertension, primary amenorrhea and lack of pubertal development. Blood evaluation revealed hypokalemia, and a low cortisol level with an increased adrenocorticotropic hormone concentration. The renin activity and testosterone and estrogen levels were suppressed, and the gonadotropin levels were high. CT scan showed bilateral adrenal hyperplasia. Besides, this patient had hyperglycemia, hyperinsulinism and negative diabetes type 1 related antibodies. A homozygous mutation c. 985 to 987delinsAA in exon 6 was found in the patient which caused the missense mutation (p.Y329fs).

**Diagnoses:**

17α-hydroxylase/17, 20-lyase deficiency combined with T2DM was considered.

**Interventions:**

The patient received dexamethasone, estradiol valerate, metformin, amlodipine besylate and D3 calcium carbonate tablets. The doses of dexamethasone was changed according to her blood potassium levels.

**Outcomes:**

After treatment, the blood pressure, blood potassium and blood glucose returned to normal range. Besides, she had restored her menstrual cycle.

**Lessons:**

For patients with hypertension, hypokalemia and lack of pubertal development, the possibility of 17OHD should be considered. The subsequent treatment would be challenging in patients with combined 17OHD and T2DM, considering the potential contribution of glucocorticoids to diabetic balance and osteoporosis.

## 1. Introduction

Congenital adrenal hyperplasia (CAH) is a cluster of autosomal recessive genetic disease caused by enzyme defects in the synthesis of steroids, which lead to disorders in cortisol synthesis. It mainly includes 21 hydroxylase deficiency, 11β-hydroxylase deficiency, 17α-hydroxylase/17, 20-lyase deficiency (17OHD) and congenital lipoid adrenal hyperplasia, among which 17OHD accounts for approximately 1%.^[1]^ 17OHD is caused by mutation of the coding gene CYP17A1, which belongs to the member of 17 subfamily of cytochrome P450 superfamily (P450c17). The encoded P450c17 have both 17α-hydroxylase and 17,20-lyase activities.^[2–4]^ During the synthesis of steroid hormones, 17α-hydroxylase catalyzed pregnenolone and progesterone, producing 17-hydroxypregnenolone and 17-hydroxyprogesterone (17-OHP) respectively, which are crucial for the synthesis of cortisol and sex steroids. 17,20-lyase is the key enzyme needed for dehydroepiandrosterone synthesized from 17-hydroxypregnenolone and androstenedione synthesized from the 17-OHP, which are the major precursors of the sex steroids testosterone and estrogen. As a result, the notable feature of patients with 17OHD are low blood levels of cortisol, androgen, estrogen and a compensatory high adrenocorticotropic hormone (ACTH) levels.^[5,6]^ Excessive levels of ACTH stimulate the 11-deoxycorticosterone (DOC) and corticosterone production, which have powerful mineralocorticoid activity, leading to extracellular volume expansion, hypertension, and hypokalemia.^[5–7]^

Type 2 diabetes mellitus (T2DM) is a chronic metabolic disorder, and is characterized by chronic hyperglycemia resulting from defects in insulin secretion and/or insulin action.^[8]^ The risk for T2DM and insulin resistance have been shown to be increased in CAH but has been mainly been blamed on supraphysiological glucocorticoid replacement, even though high androgens in poorly treated 21 hydroxylase deficiency probably also increase the risk for glycemic disturbances.^[9]^ At present, few CAH case reports had ever presented the clinical coexistent of T2DM especially in 17OHD.^[10]^ In this study, we elaborated the clinical features, laboratory tests, gene sequencing and a short-time follow up of a 17OHD patient with T2DM, in order to improve the understanding of the disease.

## 2. Case presentation

A 21-year-old female presented with a sudden disability to sit-up, noted 2 months before her admission. She was admitted with a presumed diagnosis of paroxysmal paralysis. Initial work-up in a local hospital revealed hypokalemia (potassium 1.32 mmol/L) and hypertension. Repeated blood pressure (BP) measurements throughout the day, reveled persistently, elevated BP (>170/110 mm Hg). Oral and intravenous potassium supplement was used to correct hypokalemia. Levamlodipine and spironolactone were used to control high BP in the local hospital. Her symptoms improved greatly. After discharge, oral administration of 10% potassium chloride (40–60 mL per day) was administered while her blood potassium still was not corrected to normal ranging from 2 to 3 mmol/L. Because of persistent hypokalemia she was subsequently hospitalized. She had primary amenorrhea history and did not get any medical treatment so far. She had no previous history of diagnosed mental illness, special surgery or allergy history. None of her family members including her parents, grandparents, 1 younger brother, 4 uncles and 2 aunts had similar paroxysmal paralysis or known hypokalemia, diabetes and hypertension.

Physical examinations revealed that she was relatively tall and thin, with a height of 182 cm, a weight of 63 kg. Her calculated body mass index was 19.1 kg/cm^2^. Her BP was 175/117 mm Hg at admission. She had normal heart sounds with regular rhythm and her muscular power was normal. She had no breast development and a gynecological examination showed she had genital infantilism and no pubic hair. Further laboratory investigation revealed that her white blood cell count, platelet count, hemoglobin, routine coagulation tests, liver and kidney function tests, and blood lipid tests were within the normal range (Table S1, http://links.lww.com/MD/L123). Her urine analysis showed urinary glucose 3 + and urine protein 1+.

**Figure 1.**
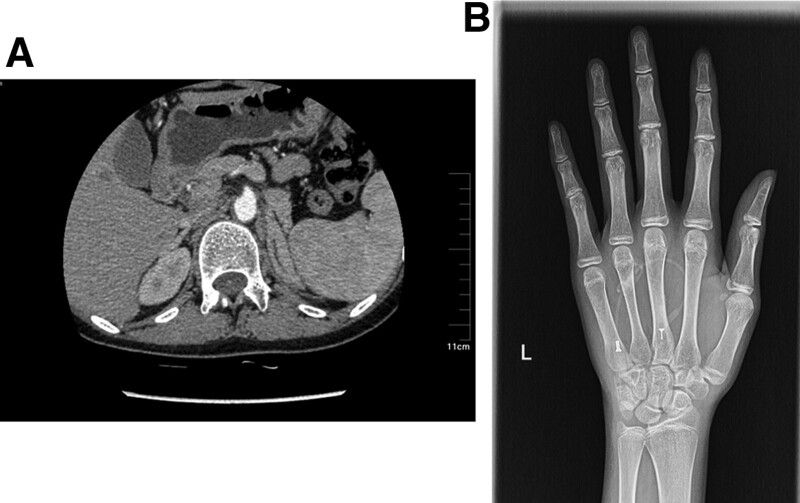
Clinical evaluations. (A) Adrenal CT showed a bilaterally longer and thicker gland. (B) X-ray imaging of carpal, phalanx and radioulnar bones.

Her serum biochemical tests revealed hypokalemia (2.11 mmol/L, normal range 3.5–5.0 mmol/L) with increased synchronous potassium excretion (69.7 mmol/L/24 h) and hyperglycemia with fasting blood glucose at 9.3 mmol/L. Blood hormone tests were checked by chemiluminescence as shown in Table 1. Her electrocardiogram showed normal sinus tachycardia and slightly ST-T changes. Pelvic ultrasound showed that her uterus was prepubertal with a size about 2.2 cm × 0.9 cm × 1.7 cm. Both her breasts and pubic hair was Tanner 1 according to the Tanner scale. She had bilateral adrenal hyperplasia especially the left side according to the adrenal computed tomography scanning (Fig. 1A). The bone mass measurement revealed she had osteoporosis with a T-score at −4.0 and Z-score at −3.8 respectively by dual-energy X-ray absorptiometry on the lumbar spine (L1–L4) and hip region. Her calculated bone age was 13-year-old according to the Greulich-and-Pyle method based on the hand X-ray (Fig. 1B), 8 years younger than her actual age. And her karyotype was normal 46, XX.

**Table 1:** Characteristics of blood hormone in the patient.

	Patient	Reference range
LH (IU/L)	31.90	Follicular phase:2–12 Luteal phase:1.0–5.0 Post-menopausal:10.9–58.6
FSH (mIU/mL)	77.69	Follicular phase: 3.3–7.9 Luteal phase:3.3–22.2 Post-menopausal:21–104
Estradiol (pg/mL)	31	Follicular phase: 40–195 Luteal phase:50–210 Post-menopausal:3.2–37
Progesterone (ng/mL)	12.6	Follicular phase: 0.2–1.2 Luteal phase:5.0–22.1 Post-menopausal:0.2–0.9
Testosterone (ng/mL)	0.11	0.15–0.51
DHEA-S (ug/dL)	4.8	18–39
SHBG (nmol/L)	24.4	18.2–135.5
Prolactin (ng/mL)	12.62	3.5–24.2
ACTH (pg/mL)	143.725	7.2–63.3
Cortisol 8:00 am (μg/dL)	0.235	4.26–24.85
Cortisol 16:00 pm (μg/dL)	0.075	2.9–17.3
Cortisol 24:00 pm (μg/dL)	0.01	lowest during the 24h
Aldosterone (pg/mL)	105.09	40–310
Plasma renin (pg/mL)	0.525	4–38
Angiotensin II (pg/mL)	88.537	49–252
Aldosterone/Renin ratio	64.30	<38

ACTH = adrenocorticotropic hormone, DHEA-S = dehydroepiandrosterone sulfate, FSH = follicle-stimulating hormone, LH = luteinizing hormone, PRA = plasma renin activity, SHBG = sex hormone-binding globulin.

Because she was newly diagnosed as diabetes during hospitalization, we did some tests to assess her blood glucose control. Her glycosylated hemoglobin at admission was 6.8% (normal range 4.27%–6.07%) and the glycosylated albumin was 22.57% (normal range 11%–16%). An oral glucose tolerance test and subsequent calculation of serum insulin were also done. The blood glucose during the oral glucose tolerance test was 8.72 to 10.37 to 14.62 to 20.00 to 21.19 mmol/L (0–30–60–120–180 minutes). The synchronous serum insulin was 43.33 to 72.33 to 83.91 to 110.2 to 126 mmol/L (0–30–60–120–180 minutes) as seen in Figure S1, http://links.lww.com/MD/L121. Diabetes-related antibodies including islet cell antibody 64KD, islet cell antibody 120kd, insulin antibody, glutamic acid decarboxylase antibody, were all negative consistent with type 2 diabetes. The urinary microalbumin was 74.31 mg/L (normal range 0–30), and urinary microalbumin/urinary creatinine was 136.40 mg/g (normal range < 30).

We sequenced a series of genes to investigate whether there were mutations in adrenal diseases-related genes and diabetes-related genes. A total of 391 candidate genes were selected (Table S2, http://links.lww.com/MD/L122&S3, http://links.lww.com/MD/L124). Six heterozygous mutations and one (CYP17A1) homozygous mutation (Table 2) was found by high-throughput sequencing. Among the heterozygous mutations, mutation in PDE11A was frameshift mutation which have been reported to be related to type 2 primary pigmented nodular adrenocortical disease, and this mutation was reported no clear significance so far.^[12]^ Mutations in RIPK4, AKR1C4, RAB3GAP2, LIPE and DMXL were probably with benign properties which do not usually cause symptoms.^[11]^ Only homozygous mutation in the CYP17A1 gene was found clinically significantly associated with 17OHD.^[13]^ Besides, we also did a multiplex ligation-dependent probe amplification of CYP17A1 gene and the results of this test showed that there were no other abnormal copies of exons.

**Table 2:** Mutations found in high-throughput sequencing.

Gene	Chromosome Location	Transcript Exon	Nucleotide Amino acid	Nature of mutation	Prediction^[11]^
CYP1 7A1	chr10:104 592420–10459242	NM_0001 02;exon6	c.985_987del TACinsAA (p.Y329fs)	Hom	D
PDE11A	chr2:178562132–178562137	NM_0169 53;exon15	c.2268_2272del (p.S757Qfs*4)	Het	-
RIPK4	chr21:43161924	NM_0206 39;exon8	c.1429A > G (p.M477V)	Het	B
AKR1C4	chr10:5248257	NM_0018 18;exon5	c.467A > T (p.D156V)	Het	B
RAB3GAP2	chr1:220330767	NM_0124 14;exon31	c.3400G > C (p.V1134L)	Het	B
_LIPE_	chr19:4290780	NM_0053 57;exon9	c.2646G > T (p.R882S)	Het	B
DMXL2	chr15:517784 212	NM_0011 74116;exon23	c.5340A > T (p.Q1780H)	Het	B

B = benign, D = detrimental, - = unknown.

**Figure 2.**
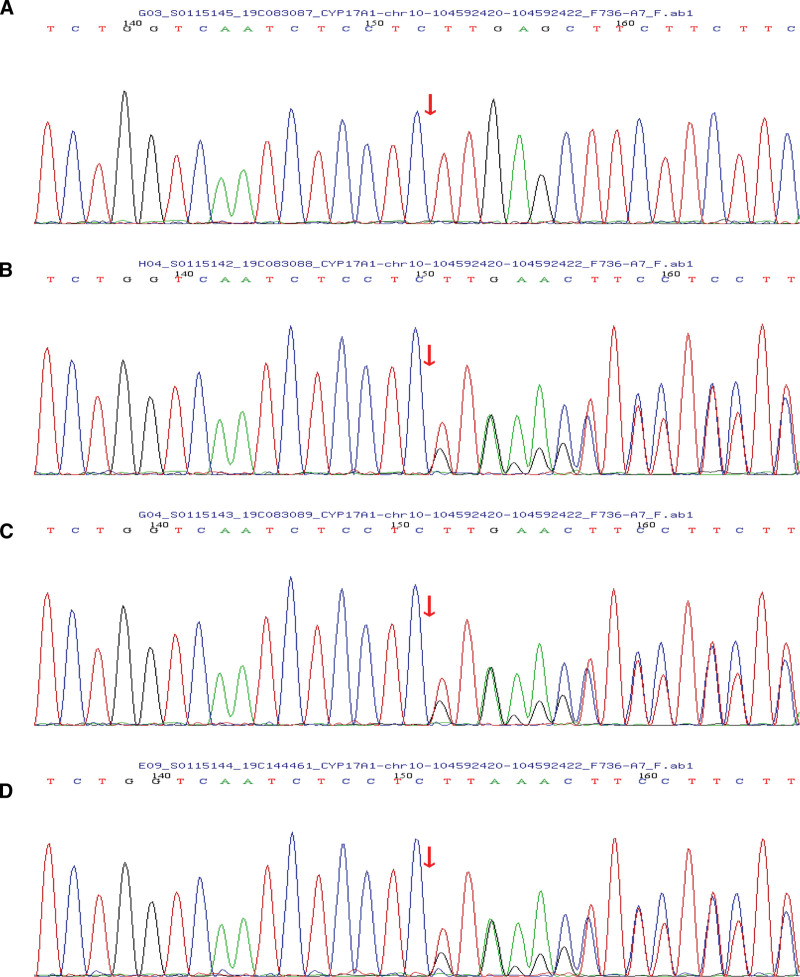
Sequencing chromatogram of CYP17A1 gene. (A) Proband. (B) Proband father. (C) Proband mother. (D) Proband brother.

**Figure 3.**
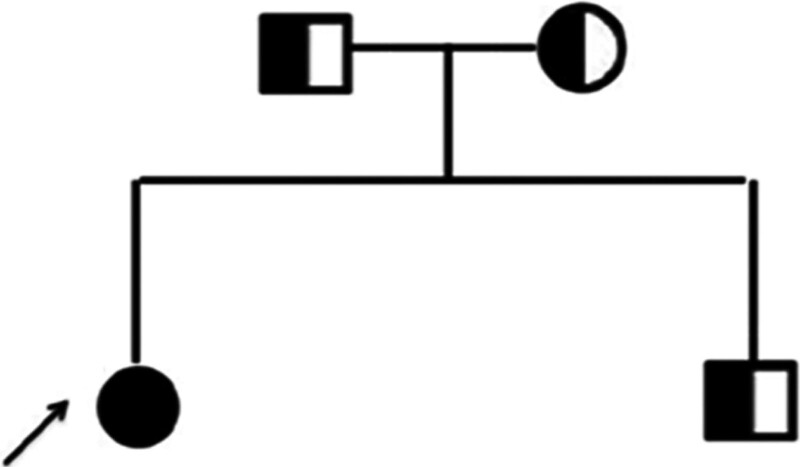
Family chart of the patient.

We chose the CYP17A1 gene mutation for further validation through Sanger sequencing. Both her mother and father had a heterozygous mutation, c.985_987delTACinsAA in exon 6 which were inherited by her at the same time. Fortunately, her younger brother did not inherit this homozygous mutation (Figs. 2 and 3).

During the follow up, we firstly prescribed dexamethasone at 0.75 mg/d, estradiol valerate at 1 mg/d, metformin 0.85 mg twice/d, amlodipine besylate 5 mg/d and D3 calcium carbonate tablets at 600 mg/d when she was discharged. After 1 month follow up, her BP fluctuated between 120 and 130/70 and 80 mm Hg, and hyperglycemia was corrected with a fasting blood glucose ranging between 4 and 6 mmol/L, and postprandial blood glucose ranging between 6 and 9 mmol/L. However, because of hyperkalemia (6.0 mmol/L), the dexamethasone dose was decreased to 0.375 mg/d. Her blood potassium returned to normal range at 3.79 mmol/L 1 week later. At the time of writing, 3 years later, her blood potassium fluctuated at normal range between 3.6 and 4.1 mmol/L when performing the yearly follow-up as shown in Table S4, http://links.lww.com/MD/L125. Besides, she menstruated twice and recently she did a pelvic ultrasound which indicated that the size of her uterus was bigger than before reaching at 3.2 cm × 1.9 cm × 2.1 cm.

## 3. Patient perspective

During hospitalization, the patient felt a significant improvement in her symptoms of limb weakness. And she reported that no similar symptoms have occurred since discharge during the follow-up 3 years. As a woman, she felt very happy to be able to resume her normal menstrual cycle and felt it very important in her life as a “complete” woman.

## 4. Discussion and conclusions

Since Yanase^[14]^ et al reported the first case of CYP17A1 gene mutation, nearly 140 mutations to date have been considered pathogenic or disease-causing in 17OHD including missense mutations, insertions, deletions and duplications, and frameshift (fs) mutations.^[15]^These gene mutations are race specific, for example, W406R mutation mostly is Hispanic, and R306C mutation mostly is Portuguese.^[16]^ Haplotype analysis suggests that such phenomena are related to “ancestral effect,” which means in some autosomal recessive genetic diseases, a few heterozygous “ancestors” carry a certain pathogenic gene, increasing the homozygous frequency and the disease prevalence through relatively limited intermarriage.^[15,17,18]^ In Chinese population, main part of 17OHD patients carried deletion mutations combined with substitution mutations. The exons 8 and 6 are the areas with high mutation incidence.^[3]^ In our study, this patient was found a homozygous mutation c.985_987delinsAA existing in exon 6 of CYP17A1 gene, which was first reported in Korean population,^[19]^ and then found very common in Chinese population.^[15]^ The nucleotide 985 to 987 (TAC) of CYP17A1 was replaced by AA in exon 6 via 985 T > C and 987delC to form a deletion/insertion mutation, causing a frameshift with tyrosine (Y)-329-Lysine(K) and the premature stop codon of 418TGA subsequently. Then a shortened protein with 417 amino acids was formed with absence of a heme-binding region (435–455 aa) which was the most important functional region of P450c17 encoded enzyme.^[20]^

Of note, besides 17-OHD, this patient was found to have a newly diagnosed T2DM considering that she had negative islet related antibodies, relatively high serum insulin level and the effective response to metformin. The relationship between T2DM and CYP17A1 gene mutations is still unclear. Wang et al reported the relationship between CYP17A1 gene polymorphism and T2DM, and proposed rs17115149 and rs12413409 polymorphisms in CYP17A1 gene were associated with T2DM in the Han population.^[10]^ Further analysis showed that rs17115149 was associated with T2DM in the hypertension subgroup, while rs12413409 was associated with high fasting blood glucose level. In pathophysiology aspect, usually 17 OHD patients may have abnormal elevation of mineralocorticoid precursors such as DOC and 18-hydroxycorticosterone. DOC can be combined with mineralocorticoid receptor, which is the core player of metabolic disease. Excessive activation of mineralocorticoid receptor can promote inflammation and insulin resistance which might ultimately lead to T2DM.^[21]^

In this case, we also sequenced 115 diabetes-related genes to explore whether some other genetic mutations contributed hyperglycemia. Although no detrimental genes were found, we found 2 heterozygous gene mutations in this patient as seen in the Table 2. One gene was the LIPE gene which encodes a key enzyme for lipolysis named hormone-sensitive lipase. Actually, impaired lipolysis has been reported as a pathogenic factor contributing to T2DM.^[22]^ Klannemark et al carried out a population and intrafamily association study in individuals with metabolic syndrome and found disequilibrium of LIPE marker in the hormone-sensitive lipase gene possibly accounting for increased susceptibility to T2DM.^[22]^ Besides, Albert et al reported that the carriers of the LIPE mutation had hepatic steatosis, dyslipidemia, systemic insulin resistance and diabetes.^[23]^ Another heterozygous gene mutation was DMXL2 gene encoding a synaptic protein named rabconnectin-3a, which was widely expressed in the brain and in the ends of the axons of neurons that produce gonadotropin-releasing hormone. Tata et al reported that DMXL2 knockdown in an insulin-secreting cell line resulted in significantly higher levels of basal insulin secretion which showed the involvement of DMXL2 in the control of insulin secretion.^[24]^ However, these reports were preliminary findings, and it was unfortunately that we did not perform a whole exome sequencing including testing the polymorphisms in CYP17A1 gene. Actually, the inner association between these 17OHD and/or CYP17A1 gene mutations and T2DM were unknown. More efforts in a larger number of patients are required to investigate the association between 17OHD and T2DM.

The main treatment for 17-OHD is glucocorticoid replacement therapy as considering its underlying pathophysiology.^[1,25]^ An appropriate dose of glucocorticoid not only inhibits the levels of ACTH and decreases the levels of mineralocorticoid precursors, but also may affect bone and carbohydrate metabolisms. There are many pharmacotherapy options such as hydrocortisone, prednisone and dexamethasone in clinic. Considering the relatively short half-life of hydrocortisone and prednisone and their effect on water and sodium retention, here we chose dexamethasone which not only could continuously inhibit the level of ACTH but also had the minimal effect on retention of water and sodium. However, Han et al reported that CAH patients on receiving dexamethasone had greater insulin resistance compared with those receiving hydrocortisone or prednisolone,^[26]^ which brought extra challenge in the glucose management in this patient with T2DM. Besides, A recent meta-analysis in CAH has shown that dexamethasone seems to have more metabolic and bone disadvantages than hydrocortisone.^[27]^ Thus, a long time follow up was required although in the previous 4 months her blood glucose was controlled well. Sometimes, even bilateral adrenalectomy might be considered if the conservative approaches continued failed.^[28]^ Besides, it should be very careful for titrating of the doses of glucocorticoid initially because if the inhibition was complete, some patients also had a rebound of potassium up to 5 to 6 mmol/L which was also happened in our case.

The growth and development of bone and the increase of bone mass are greatly influenced by steroid hormones, especially gonadal hormones. The patient was taller than average same age group, and she had osteoporosis. The delay of epiphyseal closure, the disorder of bone formation and the decrease of bone mass might be caused by the decrease of steroid hormones, especially gonadal hormones. Kardelen et al reported 6 patients with 17OHD and 5 of them found osteoporosis.^[29]^ Besides, as glucocorticoids would be taken for a long time, it would also aggravate the loss of bone mass and increase the prevalence of osteoporosis.^[30]^ Therefore, this patient gonadal steroid replacement and supportive therapy was particularly important not only for the development of secondary sexual character but also for the buildup of the bone mass.

In summary, we reported a 46, XX 17OHD patient with a c.985_987delinsAA mutation in her CYP17A1 gene combined with a coexisting T2DM. Although the inner association between these17OHD and T2DM were unknown, we found 2 other heterozygous gene mutations in this patient, LIPE and DMXL2, which might be involved in the pathology of hyperglycemia. Glucocorticoid therapy requires cautious dosing and follow-up to prevent osteoporosis and impaired glucose metabolism.

## Acknowledgments

Written informed consent was obtained from the patient for participating in the study and for publication of this manuscript. We thank the patient and her family for participating in this study.

## Author contributions

**Conceptualization:** Yumin Zhang, Yuexing Yuan.

**Data curation:** Yumin Zhang, Yuexing Yuan.

**Formal analysis:** Yumin Zhang.

**Methodology:** Yumin Zhang, Yuexing Yuan.

**Writing – original draft:** Yumin Zhang.

**Writing – review & editing:** Yumin Zhang.

## Supplementary Material










