# Supplementary material for: Genetic diagnosis and clinical analysis of 17α-hydroxylase/17, 20-lyase deficiency combined with type 2 diabetes mellitus: A case report
Source: Medicine (Baltimore). 2023 Dec 29;102(52):e36727. doi: 10.1097/MD.0000000000036727 (PMC10754554; doi:10.1097/MD.0000000000036727)
Supplement: Supplementary file 1 [file medi-102-e36727-s001.docx]

**Supplement materials**

**Table S1. Baseline characteristics of the patient.**

|  | Patient | Reference range |
| --- | --- | --- |
| **Peripheral blood** |  |  |
| WBC (10^9^/L) | 6.66 | 3.5-9.5 |
| RBC (10^12^/L) | 4.82 | 3.8-5.1 |
| Hb (g/L) | 137 | 115-150 |
| PLT (10^9^/L) | 250 | 125-350 |
| **Urinary test** |  |  |
| Glucose | 3+ | - |
| Protein | 1+ | - |
| Bilirubin | - | - |
| Urobilinogen | normal | normal |
| Ketone body | - | - |
| proportion | 1.015 | 1.01-1.03 |
| PH value | 7.5 | 5.0-8.0 |
| Nitrite | - | - |
| Occult blood | 0 | - |
| **Fecal test** |  |  |
| Occult blood | negative | negative |
| Egg | negative | negative |
| Fungus | negative | negative |
| Fat globule | negative | negative |
| **Blood** **electrolytes** |  |  |
| Sodium (mmol/L) | 142.6 | 137-147 |
| Chloride (mmol/L) | 96.3 | 99-110 |
| Phosphorus (mmol/L) | 1.26 | 0.85-1.51 |
| Calcium (mmol/L) | 2.41 | 2.11-2.52 |
| **Blood biochemistry** |  |  |
| BUN (mmol/L) | 5.4 | 2.6-7.5 |
| Creatinine (umol/L) | 57 | 41-73 |
| ALT (U/L) | 30 | 7-40 |
| AST (U/L) | 30 | 13-35 |
| ALP (U/L) | 90 | 35-100 |
| GGT (U/L) | 40 | 7-45 |
| LDH (U/L) | 239 | 120-250 |
| **Blood lipids** |  |  |
| Triglycerides (mmol/L) | 1.36 | 0.40-1.53 |
| TC (mmol/L) | 4.85 | 2.86-6.10 |
| LDL-C (mmol/L) | 2.91 | 0.00-3.12 |
| HDL-C (mmol/L) | 1.49 | >0.90 |
| **Blood fibrinolytic function** |  |  |
| PT (s) | 12.4 | 9.6-13.7 |
| APTT (s) | 33.2 | 20-40 |
| INR | 1.16 | 0.8-1.2 |
| FDP (mg/L) | 1.07 | 0-5 |
| D-Dimer (ug/L) | 38 | 0-500 |

WBC: white blood cell count; RBC: red blood cell count; Hb: hemoglobin; PLT: platelets count FDP: UA: uric acid, TC: total cholesterol, LDL-C: low density lipoprotein-cholesterol, HDL-C: high density lipoprotein-cholesterol, BUN: blood urea nitrogen, ALT: alanine aminotransferaerase, AST: aspartate aminotransferase, ALP: alkaline phosphatase, GGT: gamma glutamyl transferase, LDH: lactic dehydrogenase, PT: thromboplastin time; APTT: activated partial thromboplastin time; INR:international normalized ratio; FDP:Fibrin
