# Supplementary material for: Genetic diagnosis and clinical analysis of 17α-hydroxylase/17, 20-lyase deficiency combined with type 2 diabetes mellitus: A case report
Source: Medicine (Baltimore). 2023 Dec 29;102(52):e36727. doi: 10.1097/MD.0000000000036727 (PMC10754554; doi:10.1097/MD.0000000000036727)
Supplement: Supplementary file 2 [file medi-102-e36727-s002.docx]

**Supplement materials**

**Figure S1 Oral glucose tolerance test**
