# Supplementary material for: Genetic diagnosis and clinical analysis of 17α-hydroxylase/17, 20-lyase deficiency combined with type 2 diabetes mellitus: A case report
Source: Medicine (Baltimore). 2023 Dec 29;102(52):e36727. doi: 10.1097/MD.0000000000036727 (PMC10754554; doi:10.1097/MD.0000000000036727)
Supplement: Supplementary file 3 [file medi-102-e36727-s003.docx]

**Supplement materials**

**Table S2. Genes associated with adrenal disease**

| A2ML1 | AAAS | AARS2 | ABCD1 | AIP | AIRE | AKR1C2 | AKR1C4 |
| --- | --- | --- | --- | --- | --- | --- | --- |
| AMH | AMHR2 | ANOS1 | APC | AQP2 | AR | ARL6 | ARMC5 |
| ARNT2 | ARX | ATRX | AURKC | AVP | AVPR2 | BBS1 | BBS10 |
| BBS12 | BBS2 | BBS4 | BBS5 | BBS7 | BBS9 | BMP15 | BMP4 |
| BMPR1B | BRAF | BSND | BTK | CASR | CATSPER1 | CBX2 | CCDC28B |
| CD96 | CDKN1B | CDKN1C | CDON | CEP19 | CEP290 | CFTR | CHD7 |
| CHEK2 | CHRM3 | CLCNKA | CLCNKB | CLPP | CYB5A | CYP11A1 | CYP11B1 |
| CYP11B2 | CYP17A1 | CYP19A1 | CYP21A2 | DAZL | DCAF17 | DHCR7 | DHH |
| DIAPH2 | DISP1 | DMRT1 | DPY19L2 | DUSP6 | ERCC6 | ERCC8 | ESR1 |
| FEZF1 | FGD1 | FGF17 | FGF8 | FGFR1 | FGFR2 | FIGLA | FLRT3 |
| FMR1 | FOXL2 | FSHB | FSHR | GATA4 | GDNF | GH1 | GH2 |
| GHR | GHRH | GHRHR | GHSR | GK | GK2 | GLCCI1 | GLI2 |
| GLI3 | GNAI2 | GNAS | GNRH1 | GNRHR | GOPC | GPR101 | H19 |
| H6PD | HARS2 | HCCS | HDAC8 | HESX1 | HFE | HFM1 | HGF |
| HOXA13 | HS6ST1 | HSD11B1 | HSD11B2 | HSD17B3 | HSD17B4 | HSD3B2 | IARS2 |
| ICK | IGSF1 | IL17RD | INSL3 | INSR | IRF6 | KCNJ1 | KCNJ5 |
| KCNQ1OT1 | KDM6A | KIF1B | KISS1 | KISS1R | KLHL10 | KMT2D | KRAS |
| LARS2 | LEPR | LHB | LHCGR | LHX3 | LHX4 | LZTFL1 | MAMLD1 |
| MAP2K1 | MAP2K2 | MAP3K1 | MAX | MC2R | MCM4 | MCM9 | MED12 |
| MEN1 | MID1 | MKKS | MKRN3 | MKS1 | MRAP | MYH8 | NAA10 |
| NANOS1 | NF1 | NFKB2 | NNT | NOBOX | NR0B1 | NR3C1 | NR5A1 |
| NRAS | NSDHL | NSMF | ORC1 | OTX2 | PAX6 | PCNT | PCSK1 |
| PDE11A | PDE8B | PEX1 | PEX10 | PEX12 | PEX13 | PEX14 | PEX19 |
| PEX2 | PEX26 | PEX3 | PEX5 | PEX6 | PHF6 | PLAU | POF1B |
| POLR3A | POLR3B | POMC | POR | POU1F1 | PRKACA | PRKAR1A | PRKCA |
| PROK2 | PROKR2 | PROP1 | PSMC3IP | PTCH1 | PTPN11 | RAB23 | RAB3GAP2 |
| RAF1 | RASA2 | RBM28 | REN | RET | RIPK4 | RIT1 | RNF216 |
| ROR2 | RSPO1 | RXFP2 | RXRA | RXRB | SDCCAG8 | SDHB | SDHC |
| SDHD | SEMA3A | SEMA3E | SHH | SHOC2 | SIX3 | SLC12A1 | SLC26A8 |
| SOS1 | SOX10 | SOX2 | SOX3 | SOX9 | SPATA16 | SPRY4 | SRD5A2 |
| SRY | STAG3 | STAR | STAT5B | SYCP3 | TAC3 | TACR3 | TAF4B |
| TBX19 | TGIF1 | THRA | THRB | TMEM127 | TMEM67 | TP53 | TRH |
| TRHR | TRIM32 | TSPYL1 | TTC8 | TWNK | TXNRD2 | USP9Y | UTY |
| VHL | WDPCP | WDR11 | WNK1 | WNK4 | WNT3 | WNT4 | WNT5A |
| WT1 | ZFPM2 | ZIC2 | ZMYND15 |  |  |  |  |
