# Supplementary material for: Genetic diagnosis and clinical analysis of 17α-hydroxylase/17, 20-lyase deficiency combined with type 2 diabetes mellitus: A case report
Source: Medicine (Baltimore). 2023 Dec 29;102(52):e36727. doi: 10.1097/MD.0000000000036727 (PMC10754554; doi:10.1097/MD.0000000000036727)
Supplement: Supplementary file 4 [file medi-102-e36727-s004.docx]

**Supplement materials**

**Table S3. Genes associated with diabetes**

| ABCC8 | ACE | AGPAT2 | AIP | AKT2 | ALG3 | ALMS1 | APPL1 |
| --- | --- | --- | --- | --- | --- | --- | --- |
| ARL6 | ARMC5 | BBIP1 | BBS10 | BBS12 | BBS2 | BBS5 | BBS7 |
| BBS9 | BLK | BSCL2 | CAPN10 | CAV1 | CAVIN1 | CCR5 | CDKAL1 |
| CEL | CEP19 | CEP290 | CIDEC | CISD2 | CNBP | CTLA4 | DCAF17 |
| DMXL2 | DNAJC3 | DYRK1B | EIF2AK3 | ENPP1 | EPO | FOXP3 | GATA4 |
| GATA6 | GCGR | GCK | GLIS3 | GLUD1 | GNAS | GPD2 | HADH |
| HFE | HMGA1 | HNF1A | HNF1B | HNF4A | IER3IP1 | IFT27 | IL1RN |
| IL2RA | INS | INSR | IRS2 | KCNJ11 | KLF11 | LIPC | LIPE |
| LMNA | LMNB2 | LZTFL1 | MAFA | MAPK8IP1 | MKS1 | MNX1 | MT-TE |
| MT-TK | MT-TL1 | MTTP | NEUROD1 | NEUROG3 | NKX2-2 | OCRL | PAX4 |
| PAX6 | PDE8B | PDX1 | PLAGL1 | PLIN1 | POLD1 | PON1 | PPARG |
| PRKAR1A | PTF1A | PTRF | RETN | RFX6 | SDCCAG8 | SIAE | SLC16A1 |
| SLC16A11 | SLC19A2 | SLC2A2 | SLC30A8 | SOD2 | SPINK1 | STAT3 | SUMO4 |
| TBC1D4 | TCF7L2 | TRIM32 | TTC8 | UCP2 | VEGF | VEGFA | WDPCP |
| WFS1 | ZFP57 | ZMPSTE24 |  |  |  |  |  |
