# Supplementary material for: Genetic diagnosis and clinical analysis of 17α-hydroxylase/17, 20-lyase deficiency combined with type 2 diabetes mellitus: A case report
Source: Medicine (Baltimore). 2023 Dec 29;102(52):e36727. doi: 10.1097/MD.0000000000036727 (PMC10754554; doi:10.1097/MD.0000000000036727)
Supplement: Supplementary file 5 [file medi-102-e36727-s005.docx]

**Supplement materials**

**Table S4. Timeline**

| 2020-01-02 | A 21-year-old female presented with a sudden disability to sit-up, noted two months before her admission. |
| --- | --- |
| 2020-02-01 | She was diagnosed as diabetes, hypertension, hypokalemia, and primary amenorrhea during the during hospitalization. |
| 2020-03-31 | CYP17A1 homozygous mutation was found by high-throughput sequencing which indicated she was a 17α-Hydroxylase deficiency patient. |
| 2020-09-01 | Blood pressure, blood potassium, menstruation, and blood glucose returned normal during the follow-up. |
| 2021-09-03 | Blood pressure, blood potassium, menstruation, and blood glucose returned normal during the follow-up. |
| 2022-09-01 | Blood pressure, blood potassium, menstruation, and blood glucose returned normal during the follow-up. |
| 2023-09-02 | Blood pressure, blood potassium, menstruation, and blood glucose returned normal during the follow-up. |
